# Old disease—New reflections: Gaucher, immunity, and inflammation

**DOI:** 10.1111/jcmm.70087

**Published:** 2024-10-27

**Authors:** Can Veysel Şoroğlu, Ezgi Gizem Berkay

**Affiliations:** ^1^ Department of Medical Biotechnology Acıbadem Mehmet Ali Aydınlar University, Institute of Health Sciences Istanbul Turkey; ^2^ Department of Basic Sciences, Dentistry Faculty Istanbul Kent University Istanbul Turkey

**Keywords:** COVID‐19, Gaucher disease, Glucocerebrosidase, immunity, neuroinflammation

## Abstract

Gaucher disease (GD) is the most common lysosomal storage disease. It is a multisystemic metabolic disease caused by *GBA* pathogenic mutations. Although the general symptoms have been known for a long time, new treatment possibilities, the detection of different biomarkers, and innovations in diagnosis and follow‐up have paved the way for further studies. Recent studies have shown that the immune system has become an essential factor associated with disease progression. The role of Gaucher cells in the disease is well characterized. In addition to phagocytic macrophage cells, lymphocytes, complement system, and inflammatory pathway elements are also implicated in GD as they were shown to be the underlying factors causing associated pathologies such as Parkinson's. In this article, the relationship between the GD and the immune system has been examined and reviewed in light of new findings.

## 1 INTRODUCTION

Gaucher disease (GD) is a well‐known, rare, inherited autosomal recessive, lysosomal storage disease with specific clinical and biochemical findings. GD is characterized by the accumulation of glucocerebroside (Glucosylceramide, GlcCer) as a result of a deficiency in β‐glucosidase enzyme activity (Glucocerebrosidase, GCase, EC: 4.2.1.25, an acid hydrolase), that is involved in the degradation of glycosphingolipids.^1^ The multisystemic effects modulate the immune system due to biallelic mutations in the *GBA1* gene (1q22), which causes the main pathological events associated with sphingolipid accumulation in monocyte–macrophage‐derived cells. In this review, we aim to shift the focus to the immunological aspects of GD, a relatively unexplored area in the field. While most studies have concentrated on the genetic elements and molecular pathways leading to GD, understanding the immunological effects is equally crucial and can provide new insights into the disease.

Classification of GD mostly depends on the symptoms, onset of disease, and manifestation in different tissues. Diminished neuronal cells and increased activation of glial cells can be seen in neuronopathic GD patients.^2^, ^3^ GD represents a spectrum of diseases from perinatal‐lethal to non‐neuronopathic mild form. GD Type 1 (non‐neuronopathic, GD1; OMIM 230800) is the most prevalent form of lysosomal storage diseases (LSDs) and GD. GD Type 2 (acute neuronopathic, GD2) and Type 3 (sub‐acute neuronopathic, GD3) correspond to the rest of the cases.^4^

Even the classification of GD takes form by the affected tissue types; the underlying events in GD arise from macrophages, and activity loss in the cellular enzyme occurs, leading to the accumulation of glucocerebroside and similar molecules, thereby affecting the lysosomal functional capacity. In macrophages, mutated forms of GCases (lysosomal, non‐lysosomal, and cytosolic GCase) end with the accumulation of glucocerebrosides within the cell. While the observed phenotype occurs mainly due to enzyme deficiency, intracellular metabolic activities are affected first, and then phagocytic immune response disrupts the functions of related cells. However, there is no clear evidence of whether both lysosomal and non‐lysosomal enzyme activities play a role in the Gaucher phenotype.

The GCase enzyme uses glycosphingolipids as a substrate. Glycosphingolipids' macromolecular structure allows them to be utilized as a cell membrane component, playing a role in recognizing bacteria and toxins and participating in physiological processes and signal transduction. Enzyme defects in glycosphingolipid metabolism cause an imbalance and deficiency in the aforementioned functions.^5^

As the accumulation of glucocerebrosides continues, the growing and thickening lysosome structure forces the cell to change similarly. As a result, reticuloendothelial cells transform into Gaucher cells. Again, the increasing amounts of glucocerebroside in the circulation are taken up by phagocytes. Their cytoplasm transfigures into a form called “wrinkled tissue paper,” which is a pathognomonic appearance for these cells.^6^

The manifestations of GD in different tissues are based on the various types of tissue macrophages. Various examples of how tissue involvement shapes GD prognosis are reported. Since haematopoiesis requires special microenvironmental areas (niches) in the bone tissue, as in the example of osteoclasts, bone involvement of GD ends up with an increased immune response and an elevated amount of plasmacytoid and myeloid dendritic cells in GD cases.^7^ Osteoclasts, part of the phagocytic cell population, cause bone resorption due to tartrate‐resistant acid phosphatase (TRAP) enzymes. Due to these activities, they are seen as one of the leading actors in diseases with bone destruction.^5^ Studies with animal models showed that osteoclasts did not differ in the presence of GCase mutations.^8^ In addition, GD prognosis is also associated with neurodegenerative diseases, particularly Parkinson's, autoimmune diseases, blood cell disorders, and haematological malignancies like multiple myeloma.^9^

### 1.1 Gaucher, macrophages and phagocytosis

**FIGURE 1:**
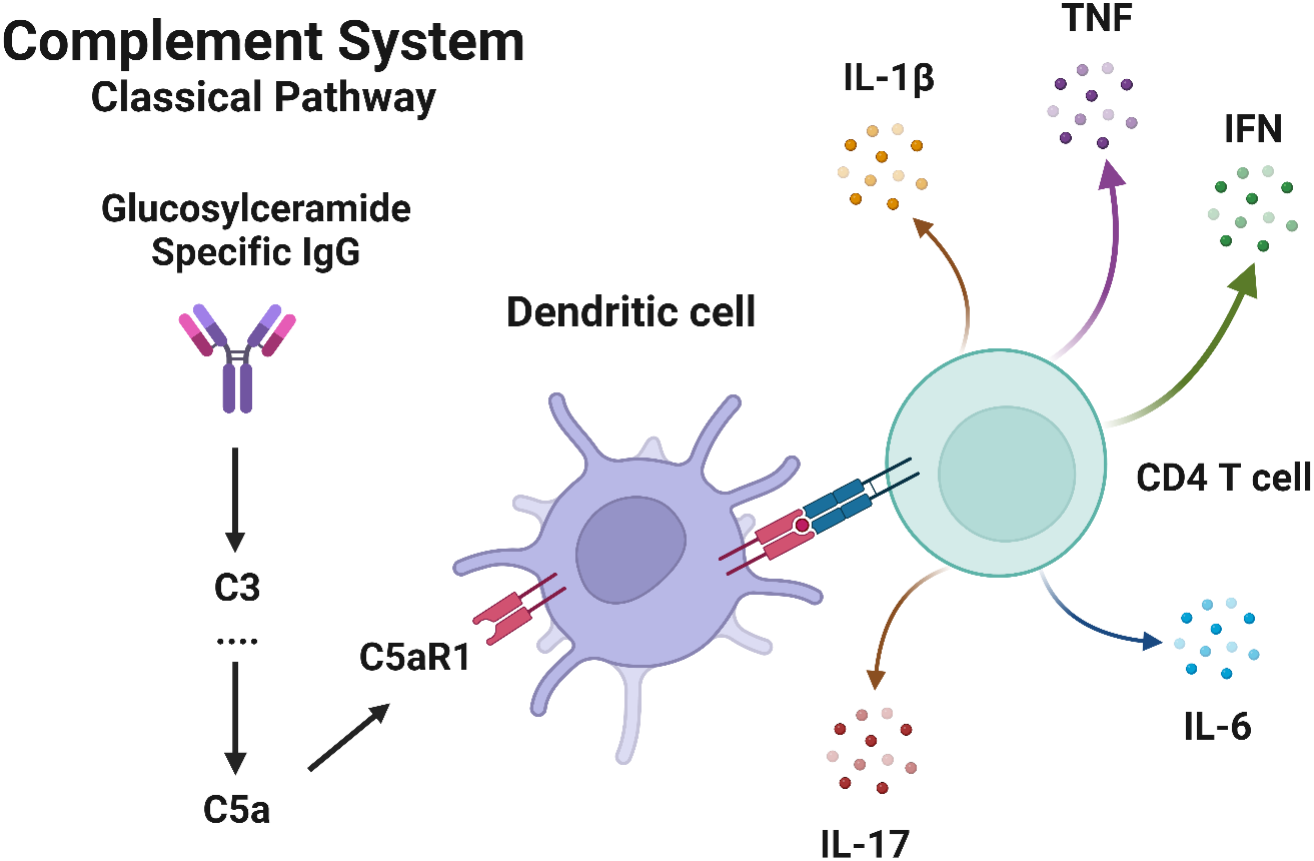
The effect of normal and defectly folded enzymes on lysosomal degradation. The failure of phagosome formation and its effect on lysosomal degradation (Created with BioRender.com).

The lysosomal recycling process should be understood clearly to understand the cellular mechanisms in GD. The initial step of endocytosis is forming a plasma membrane‐derived endocytic vesicle and fusion with an early endosome. In the meantime, phagophores take shape in the cytoplasm to fuse with the late endosome (LE). Together, they form the autophagosome, a spherical bilayer membrane, and the leading figure of macroautophagy. Sooner, this fusion vesicle becomes LE and merges with the outer membrane of the autophagosome in lysosomes, and the complex will be ready for the autophagy process.^10^ When the effects of GD on phagocytosis were investigated, it was observed that the recognition and uptake of apoptotic cells by macrophages were as accurate as healthy cells, and there were irregularities during the digestion processes caused by the failure of the phagosome to mature and fuse with the lysosome.^11^ Normal and defective pathways were schematised in Figure 1.

In a study on a neuronopathic mouse model, nonfunctional autophagosomes were also accumulated in addition to cellular storage of glucosylsphingosine and glucosylceramide.^12^ Neurons and astrocyte cultures derived from mice deficient in *Gba*, *Psap*, or glycoceramidase enzymes and brain cells from these models indicate the presence of accumulated autophagic cargo, including dysfunctional mitochondria ubiquitin labelled protein aggregates, α‐synuclein, and p62 with autophagosomes and lysosomes.^13^, ^14^, ^15^, ^16^ Lysosomal errors, GCase accumulation, and autophagy disruption have also been associated with reduced survival, neuron destruction, and locomotor system defects in the *Gba* gene knockout Drosophila model.^17^

Although it was not common in the literature, GD's erythrophagocytic activity was first described in 1967 by observing stained iron particles of the phagocyted erythrocytes.^18^, ^19^ Defective sphingolipids accumulate in macrophages and erythroid progenitors at the erythropoiesis step and mature red blood cells in the peripheral circulation. As a result of this accumulation, the life cycle of erythrocytes is shortened, and their phagocytosis increases. Meanwhile, digesting erythrocytes, tube‐like formations appeared in cells that will transform into Gaucher cells.^20^ Bone marrow examination in an adult GD case revealed that ~15% of Gaucher cells showed erythrophagocytic activity.^21^ This rare hemophagocytic lymphohistiocytosis (HLH) syndrome has also been reported in a 5.5‐month‐old GD case. HLH is a fatal syndrome derived from the uncontrolled production of inflammatory cytokines by cytotoxic T lymphocytes and histiocytes.^22^

### 1.2 Inflammation in Gaucher disease

Multisystemic manifestations of GD may primarily arise from the combined effects of the exponential accumulation of lipid molecules in the lysosome, causing multiple inflammatory responses and dysfunctions of autophagy mechanisms. Both mutational background (or genetic heterogeneity) and other affected cellular activities that affect the degree of decrease in enzyme activity shape disease progression in a patient. Even in cases with a moderate phenotype, it's accepted that there's a minimum inflammatory response.^23^

The innate immune responses prevent pathogens from entering the organism and are orchestrated by a plethora of mediator molecules, such as TNF, IL‐1, IL‐6, IL‐10, IL‐12, IL‐15, IL‐18, IFN‐α, IFN‐β, and IFN‐ɣ. A secondary reply specific to the antigen develops with memory cells that recognize the antigen, proliferation, and differentiation in the acquired immune response. It is divided into two subgroups: Humoral (B lymphocyte/antibody‐mediated) and Cellular immunity (T lymphocyte‐mediated). Following both the increased frequency of immunologic events and elevated ratio of immunological cells in circulation, many inflammatory factors such as I‐309, CXCLs, and MCP‐5, HGF, TNF‐α, TGF‐β1, MCSF, MIP‐1, CCL18 and IL‐1α, IL‐1β, IL‐1Ra, IL‐2R, IL‐6, IL‐8, IL‐10, IL‐18 become activated.^24^

In macrophages with a pathogenic genomic variant (N370S/N370S) of Gaucher patients, activating the inflammasome leads to increasing proinflammatory IL‐1β and IL‐6 release and impaired autophagy. Increased autophagic adapter p62 was found in Gaucher cells, and it has been shown that p62 activates p65‐NF‐kB in the cell nucleus.^23^ The expression levels of IL‐1 and IL‐1R antagonist, IL‐2 soluble receptor, TGF, MCSF, and CCL‐18 were most elevated.^25^ Besides, in a study conducted with 25 controls, 13 untreated GD patients, and 49 GD patients treated with ERT, it has been determined that there is intense VD1d and MHC‐II expression in monocytes, and this situation has been associated with increased T cell activation.^26^

On the other hand, in studies conducted with cell models obtained from Gaucher patients, it was observed that increased cytokine production by osteoclasts, such as IL‐8, IL‐6, and TNF‐α, and chemokines such as MCP1 up‐regulated, which also increased the activity of osteoclasts.^27^, ^28^ It has been shown that one of the main factors inducing the differentiation of monocytes into osteoclasts is RANKL, a surface marker that belongs to the TNF protein family in differentiation, and IL‐1β, which acts synergistically with it in the patient's bone and marrow environment.^29^, ^30^, ^31^, ^32^ In addition, in vitro studies with cells from GD1 patients found a clinical correlation between osteoclast differentiation and bone mineral density.^31^, ^33^ That means osteoclasts have increased activity with disease, leading to bone mineral density and osteopenia loss.

Decreased MHC‐class II expression was found in GD patients receiving enzyme replacement therapy (ERT), in which increased chitotriosidase activity was observed in activated macrophages.^6^ While it is seen that inflammatory cytokines approach normal levels with enzyme replacement therapy,^34^ improvement is not observed for osteopenia, which is associated with high levels of IL‐6 and TNF‐α. In addition, while IL‐4 and MIP‐1α increased, anti‐inflammatory cytokines IL‐10 and IL‐13 decreased.^35^ In a study in which 26 SNPs in 14 genes associated with bone metabolism were examined with 83 cases of GD with bone involvement, variants in *BDNF, ESR1, OPN, RUNX*, and *VDR* genes were found as candidates as altering levels of cytokines and change the progress of GD.^35^ To determine the role of bone marrow MSCs in immune system involvement in GD, in a study conducted with a case diagnosed with GD type 1, the cytokine‐related gene expressions of normal and GD mesenchymal stem cells were examined, it was seen that there was an increased expression of *PGE2*, *IL‐8*, and *CCL2*, the osteoclast activating genes. According to these results, it was thought that this activity of CCL2 accelerated the transformation of monocytes into osteoclasts and was effective in bone involvement in the disease.^36^

### 1.3 Gaucher and neuroinflammation

One of the remarkable findings of recent years is the relationship between the heterozygous variants of the *GBA1* gene and a 4%–9% increase in the probability of developing Parkinson's disease.^37^ Even if they do not fully meet the criteria of GD, more than 21% of GD type 1 cases show Parkinsonian findings.^38^ Parkinson's disease, characterized by the progressive degeneration of dopaminergic neurons in the substantia nigra and the accumulation of intracellular alpha‐synuclein, is one of the critical health problems in adulthood. Activating microglia, macrophages, and lymphoid cells is one of the main pathophysiological pathways leading to this disease. T‐cell infiltration was detected in the brain tissues of the patients with Parkinson's. Also, B cell‐derived immunoglobulin G accumulation was observed.^39^, ^40^, ^41^

It has been suggested that the decrease in glucocerebrosidase activity is directly proportionate to advanced age, and age‐related neurodegeneration risk may also be associated with this condition.^42^ By examining the changes in glycosphingolipid levels in Parkinson's disease, the decrease of glucocerebrosidase and alpha‐galactosidase activity was found in the brain tissue. Similarly, the major brain sphingolipids, the GM1, GD1a, GD1b, and GT1b levels, have decreased, while GM2 and GM3 levels were increased in patients who carry mutations in the *GBA* gene. As the level of beta‐hexosaminidase decreases in the cerebrospinal fluid and increased cathepsin E and beta‐galactosidase activity were observed, increased monohexylceramides and GM3 levels were detected in the blood.^43^ The neurological involvement and relationship between GD and Parkinson's disease show that the fusion of autophagosomes with lysosomes and the degradation of protein aggregates may also be effective.^44^, ^45^

In a study conducted with neuronopathic Gaucher‐generated mice published by Vardi et al. in 2020, it was shown that GlcSer/GlcSph accumulation activates both primary and secondary pathological pathways, and the activated IFN cytokine pathway with pathogen recognition receptor (PRR) stimulation reduces neuroinflammation in nGD mice.^46^ Another study examining the interaction between nGD and neuroinvasive Sindbis virus (SVNI) infection found that infection did not affect disease severity. Still, these nGD mice were more resistant to SVNI infection. Also, the inflammatory response in neuronopathic GD *Gba* ^flox/flox^; nestin‐Cre mouse brains were examined, and it was reported that macrophages did not infiltrate, and increased activity of astrocytes and microglia was observed.^47^

### 1.4 Complement system

**FIGURE 2:**
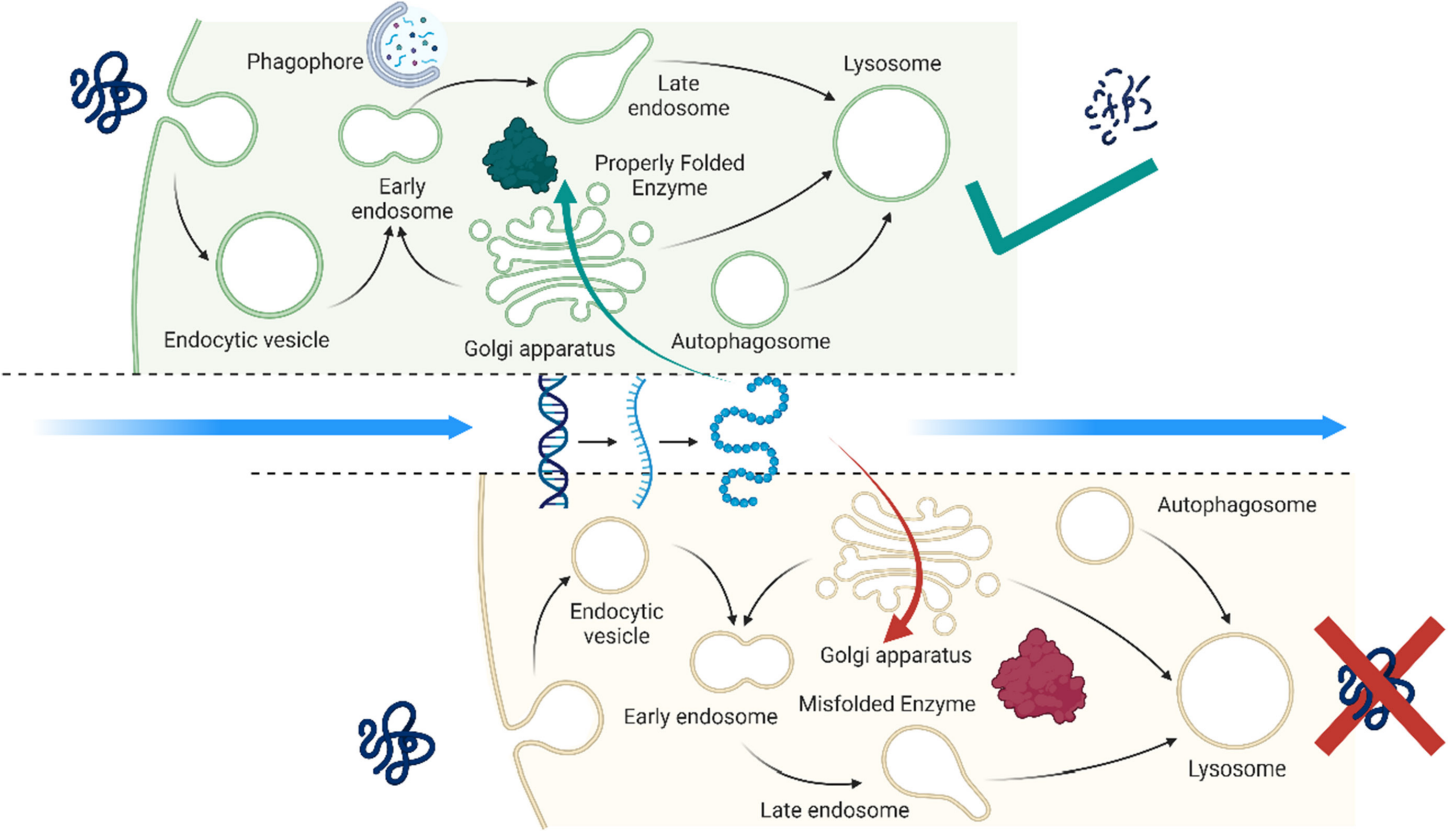
Complement System in Gaucher Disease (Adapted from “Endocytic Pathway Comparison”, by BioRender.com (2023). Retrieved from https://app.biorender.com/biorender‐templates).

The complement system drives glucosylceramide accumulation and tissue inflammation in GD.^48^ The main controlling system in the accumulation of GlcCer is the activation of complement C5a and C5aR1. GlcCer‐specific IgG molecules that activate C5a and C5aR1 are associated with UDP glucose ceramide glucosyltransferase production in GD.^48^ The key to its activity is the two “seven transmembrane domain” containing C5a receptors, C5aR1 and C5aR2. The interaction of C5a and C5aR1 in dendritic cells increases the expressions of CD40, CD80, and CD86 and stimulates CD^4+^ T cells.^49^ In studies with *Gba* knockout mice, CD40, CD80, and CD86 of dendritic cells show an increased C5a activation and increased expression of CD40L and CD69 of CD^4+^ T cells. In addition, it has been observed to stimulate the production of cytokines IFN, TNF, IL‐1β, IL‐6, and IL‐17 (Figure 2).^48^

### 1.5 Gaucher and acquired immunity

Acquired/adaptive immunity occurs with memory, responds slowly, specific for the antigen, and leads to clonal expansion. The main cell type is the memory T cells; B lymphocytes are also essential with a secondary response of IgG antibodies.

#### 1.5.1 T lymphocytes

In an immunophenotyping study conducted to detect lymphocyte subgroups in Gaucher cases, it was found that activated T lymphocytes (CD^3+^ HLA^−^ DR^+^), activated T helper, and cytotoxic T cells were increased compared to the control group.^50^

In a study conducted with mice, it was observed that CD4+ cells increased due to increased cytokine levels, and maturation in the thymus was impaired.^51^, ^52^ In addition, it was observed that the CD4/CD8 ratio decreased (normally 2/1), and T cell activation increased with the disease.^53^ A study conducted in Egypt with 20 paediatric GD patients and 20 healthy controls found that CD8+ T lymphocytes increased and NK cells decreased in GD patients.^54^

In another study with Gba knockout mice, haematopoietic stem cells and progenitor immune cells were examined, and it was reported that the most remarkable finding was observed in the thymus. These changes include impaired T‐cell maturation, defects in B‐cell recruitment, increased antigen presentation, and irregular departure of mature thymocytes from the organ. It has been said that these findings may be due to the antiproliferative effect caused by lipid accumulation and may be observed in other lipidoses.^55^ In another study, after examining cytokine arrays, microarrays, and immunophenotyping, it was reported that thymic T cell and dendritic cell development were impaired, and the function of macrophages was lost.^8^

#### 1.5.2 B lymphocytes

B1 cells, a subtype of B cells, are the main cell type in developing B‐cell chronic lymphocytic leukaemia. And in Gaucher, patients were reported to have immunoglobulin abnormalities, hypergammaglobulinemia, and plasmacytosis, too. The defects in levels of IgG and IgM and plasmacytosis are the disorders of the B cell subgroups, leading to an increased risk of having pathologies such as gammopathies and multiple myeloma.^9^ Deterioration of CD20^+^/CD27^+^ ratios indicates that memory formation of B cells is also impaired in GD cases. Therefore, a decrease in B cell levels is expected in GD cases, and this decrease could be related to chronic immune system activation and inflammation in GD cases.^38^, ^39^ In GD cases, it has been shown that the reduction in Breg cells is related to the course and severity of the disease and that there may be an increase in all GD subtypes compared to normal levels of CD19^+^ B cells.^45^

### 1.6 Natural killer cells

Even though there seems to be a trend of increased NK cells in GD, no significant change in NK cell fraction was seen between GD and control groups; CD3 expressing NK cell fraction was markedly higher in the GD group compared to non‐GD controls. In the same study, the invariant NKT cells (iNKT cells, CD3^+^, Vα24Jα18^+^, CD4^+^/CD8^+^) are a subgroup of NKT cells that express an invariant T cell receptor α‐chain, Vα24‐Jα18. Subgroups of iNKT cells expressing CD4 and CD8 are significantly increased in the GD group.^53^

### 1.7 Gaucher and autoimmunity

Autoimmunity occurs when immune tolerance is broken, and the immune system recognizes self‐antigens as foreign. In a study that aims to detect autoantibody levels in Gaucher patients (*n* = 43), a significant increase was observed in natural autoantibodies such as rheumatoid factor, anti‐ribonucleoprotein, and anti‐pyruvate dehydrogenase, which indicate possible involvement of autoimmunity in the disease pathology. Natural autoantibodies can be detected in healthy people without any tissue damage and are thought to have homeostasis functions.^56^

### 1.8 Gaucher and cancer

Reported co‐occurring malignities with GD include multiple myeloma (MM), leukaemia (acute, chronic), and Hodgkin's disease. A predisposition to malignancy in GD cases has long been known, for example, up to ~25‐fold increase in risk for developing multiple myeloma.^57^, ^58^ The underlying factor seems to be related to continuous activation of the B cells at a 1.8 times higher risk for overall cancer development and association between late‐onset GD cases and increased malignity risk, especially solid organ tumours.^9^

In addition to known immunological/inflammatory factors, mitotic activator kinases such as p38, which act as precursors/activators of these molecules, are thought to play a role in GD development. In a study, isophagomine (pharmacological chaperone) was given to the neuronopathic GD mouse model; elevated Gcase activity increased the ceramide and down‐regulated p38δ isoform via ceramide‐activated phosphatases. Combined with the inflammatory response, increased mitotic activators, the disruption of the sphingolipid cycle, and abnormal levels of cytokines provide a suitable condition for carcinogenesis. They are likely associated with increased cancer rates in GD cases.^59^, ^60^, ^61^, ^62^

### 1.9 Gaucher disease, infections, and COVID‐19

It is still necessary to explain the effects of GD in conjunction with the physiopathological mechanisms identified thus far. In some cases, there is an increased susceptibility to infection and a tendency for frequent infections. GD patients' clinical status decline can be attributed to viral infection due to their affinity for lymphoid cells, such as the Epstein–Barr virus.^63^

SARS‐CoV‐2 is another viral source that triggers abnormal levels of inflammatory cytokines, resulting in immune cell activation. In the same way as LSDs, SARS‐CoV‐2 infection is associated with lysosomal involvement and increased IL‐2R, IL‐6, IL‐10, MIP1‐α, and TNF‐α levels.^23^, ^64^, ^65^, ^66^ Spleens and hilar lymph nodes obtained from autopsies of 6 deceased COVID‐19 cases revealed that positive SARS‐CoV‐2 nucleoprotein was found in ACE2‐expressed CD68^+^ CD169^+^ macrophages.^67^

Studies conducted on Gba1 mutant mouse cells and primary cell cultures from the individuals with GD have demonstrated an increase in inflammation‐initiating molecules, specifically C5a‐C5aR1, a component of the complement system. Gain, treatments targeting the C5a‐C5aR1 pathway have been found to suppress the inflammatory response and decrease SARS‐CoV‐2 replication. As a result of these findings, a clinical interpretation was made stating that in COVID‐19 infections with or without GD, if treatment targeting the complement and glycosphingolipid pathway is combined with anti‐inflammatory medication, worsening of the prognosis or reinfection can be prevented.^68^

In a study conducted with 110 LSD patients (GD *n* = 43, MPS *n* = 55, Pompe disease *n* = 12) with a median age of 129 months and all receiving ERT, 53.6% (*n* = 56) of at least one autoimmunity or immunodeficiency‐related parameter abnormality was detected. Of these 56 patients, 23 had GD, 26 had MPS, and 7 had Pompe disease. The autoantibodies associated with autoimmunity in patients diagnosed with Gaucher were ANA, anti‐DNA, antithyroglobulin, anti‐TPO, TRAB, antiphospholipid‐IgM, and anticardiolipin IgM. However, only one GD Type 1 patient showed findings related to Hashimoto's thyroiditis, and no clinical presentation was detected in other patients. Notably, only 2 GD patients were infected with COVID‐19 but survived the disease without any sequelae, although their laboratory findings were abnormal. Twenty‐one of the 43 GD patients in the same study were evaluated for immunodeficiency. Among 21 GD patients, 12 (57%) exhibited immunodeficiency‐related parameters, with 7 Type 3 and 5 Type 1 cases. One GD Type 3 patient had a lymphocyte count <1500/mm^3^, 2 GD patients had eosinophilia, 6 GD patients had low NK cell counts, and another GD Type 3 patient had low B cell counts. Although low immunoglobulin (Ig) levels are observed among these 21 GD patients, GD patients' Ig levels are closer to normal than those of MPS and Pompe patients among all LSDs.^69^

Besides viral infections, bacterial infections also pose a risk for GD patients. Researchers found that homozygous N370S mutations in the Gba1 gene cause resistance to tuberculosis infection in zebrafish. Even low concentrations of glucosylsphingosine have been shown to inhibit mycobacterial growth in GCase‐deficient macrophages. Glucosylsphingosine accumulate in phagolysosomes, and the low concentration of the positively charged micelle formation in phagolysosomes is thought to be the underlying cause of tuberculosis resistance. The existence of heterozygous pathogenic variants in populations with frequent lysosomal storage diseases, such as Ashkenazi Jews, can be explained by heterozygous advantages and resistance mechanisms to common infectious diseases such as tuberculosis. The N370S variant is widely prevalent in this population due to multiple severe population bottlenecks and selective advantages.^70^

To better explain the effects of COVID‐19 and similar infections on the prognosis of GD and the impact of drug treatment, further studies are needed that include a larger number of GD patients.^64^, ^66^, ^67^

## 2 CONCLUSION

Gaucher cells are macrophage‐derived cells that store an extreme amount of glucocerebroside (glucosylceramide, glucosylcerebroside), a common storage material in GD, which appears after a mutation in the gene that encodes beta‐glucosidase. These lipid‐filled cells have an easily distinguished morphology and can be seen in various organs such as the spleen, liver, lung, bone marrow, central nervous system, and lymph nodes. Imbalances and deficiencies in GD pathways may also lead to an increase in immunological sequelae characterized by a marked increase in many inflammatory molecules. A thorough understanding of GD requires a detailed understanding of the pathologies such as neurodegenerative diseases, autoimmune diseases, blood cell disorders, and haematological malignancies (i.e., multiple myeloma) associated with GD. As a result, there's an urgent need to analyse and understand the shared immunological aspects underlying these pathologies.

## AUTHOR CONTRIBUTIONS

**Can Veysel Şoroğlu:** Conceptualization (equal); data curation (lead); investigation (lead); resources (lead); writing – original draft (equal). **Ezgi Gizem Berkay:** Supervision (lead); writing – original draft (equal); writing – review and editing (equal).

## FUNDING INFORMATION

The authors have no funding to report.

## CONFLICT OF INTEREST STATEMENT

The authors have no conflicts of interest to declare.

## Data Availability

Data sharing is not applicable to this article as no datasets were generated or analysed during the current study.
